# Delayed diagnosis and poor treatment compliance remain key challenges in leprosy control: a case of lepromatous leprosy with type 2 reaction

**DOI:** 10.1186/s12879-026-13257-y

**Published:** 2026-04-09

**Authors:** Maheshi Thilakarathna, Nivedha Uthayarajan, Chathurarya Siriwardena, Nirupa Pallewatte, Bhagya Deepachandi

**Affiliations:** 1 Faculty of Graduate Studies, University of Sri Jayewardenepura, Nugegoda, 10250 Sri Lanka; 2 Department of Life Sciences, Faculty of Science, NSBM Green University, Homagama, 10206 Sri Lanka; 3 Central Leprosy Clinic, The National Hospital of Sri Lanka, Colombo, 00700 Sri Lanka; 4 Anti Leprosy Campaign, Welisara, Ragama 11010 Sri Lanka

**Keywords:** Delayed diagnosis, Leprosy, *Mycobacterium leprae*, Non-adherence, Type 2 reaction

## Abstract

**Background:**

Leprosy (Hansen’s disease) is a chronic granulomatous infection primarily caused by *Mycobacterium leprae*. Since 2008, *Mycobacterium lepromatosis* has also been identified as an additional causative pathogen. Without timely treatment, the disease can progress to serious neuropathy and permanent disability. Lepromatous leprosy, the most infectious form, is associated with high bacillary load, systemic involvement, and immune-mediated complications such as type 2 reaction. Treatment with multidrug therapy is effective, but patient non-adherence remains a major challenge to disease control.

**Case presentation:**

This case report details a 39-year-old unmarried male mechanic from Colombo, Sri Lanka, diagnosed with lepromatous leprosy, characterized by facial skin and earlobe infiltration, madarosis, ichthyosis, saddle nose deformity, symmetrical skin lesions, and peripheral neuropathy. Initially diagnosed in December 2023, the patient defaulted on treatment and returned in June 2025 with worsened symptoms, including grade 2 disability and type 2 reaction. Slit skin smears revealed a persistent bacterial index of + 6, while the morphological index declined from 15% to 2%, suggesting chronic progression. Multidrug therapy was re-initiated, and type 2 reaction was managed with systemic corticosteroids and supportive physiotherapy.

**Conclusion:**

This case highlights the severe consequences of multidrug therapy non-adherence in lepromatous leprosy, including progression to type 2 reaction and disability. It reinforces the importance of prompt diagnosis, treatment persistence, patient education, and consistent follow-up to prevent complications, reduce type 2 reaction associated morbidity, and interrupt transmission. Regular follow-up is essential to ensure timely intervention and improved outcomes.

## Background

Leprosy is a chronic infectious disease primarily caused by *Mycobacterium leprae*, with *Mycobacterium lepromatosis* also recognized as a pathogen since 2008 [1]. Transmission occurs mainly via respiratory droplets from untreated individuals, particularly after prolonged close contact [2]. The disease develops insidiously, with an average incubation of five years, though symptoms may take up to 20 years to appear [3], and clinical manifestations vary based on the host immune response [4]. Multidrug therapy (MDT), combining rifampicin, dapsone, and clofazimine, remains the World Health Organization (WHO) recommended standard of care [5]. Untreated leprosy can lead to irreversible nerve damage, disabilities, and social exclusion [6].

Diagnosis relies on WHO cardinal signs: hypopigmented or reddish skin patches with sensory loss, thickened peripheral nerves with sensory or motor deficits, or the presence of acid-fast bacilli (AFB) in skin smears or biopsy specimens [7]. Leprosy is classified for treatment purposes as paucibacillary (PB) or multibacillary (MB) based on lesion count and smear results [8], and the Ridley-Jopling system categorizes disease severity from polar tuberculoid (TT) to lepromatous leprosy (LL) [6].

LL, the most infectious form, is characterized by poor cell-mediated immunity, high bacillary load [9], multiple symmetrical skin lesions, and extensive nerve involvement [10]. Clinical signs include leonine facies, madarosis, thickened earlobes, saddle nose deformity [11], ichthyosis of the lower limbs, glove-and-stocking anaesthesia, ulcers, and deformities, with male patients potentially experiencing testicular atrophy and sexual dysfunction [12, 13]. A type 2 reaction (T2R), or erythema nodosum leprosum (ENL), is an immune complex-mediated condition typically seen in LL patients with high bacterial loads [14], presenting as painful erythematous nodules with systemic symptoms [15]. In this case, the patient developed T2R accompanied by anaemia and leucocytosis, and progressive neuropathy following treatment default.

Despite advances in MDT based leprosy control, delayed diagnosis and treatment default remain significant challenges in endemic settings. Late presentation often reflects missed early detection at primary care, inadequate patient counseling, gaps in case retrieval, limited access to specialized services, poor awareness, and insufficient follow-up.

This case highlights a critical but often overlooked gap in leprosy control, emphasizing the consequences of delayed diagnosis and interruption of MDT on disease progression and transmission. It underscores the need for improved patient retention strategies, strengthened follow-up and contact tracing, and enhanced programmatic support to prevent disability and further disease progression.

## Case presentation

### Initial presentation

A 39-year-old unmarried male mechanic from Mattakkuliya, Colombo, Sri Lanka, living alone, presented to the dermatology clinic at the National Hospital of Sri Lanka (NHSL) in December 2023. Referred by a primary care physician for suspected Hansen’s disease, he reported thickened facial skin over the eyebrows, earlobes, and nose, along with multiple anaesthetic patches on both arms for over a year. No previous evaluation had been performed.

**Fig. 1:**
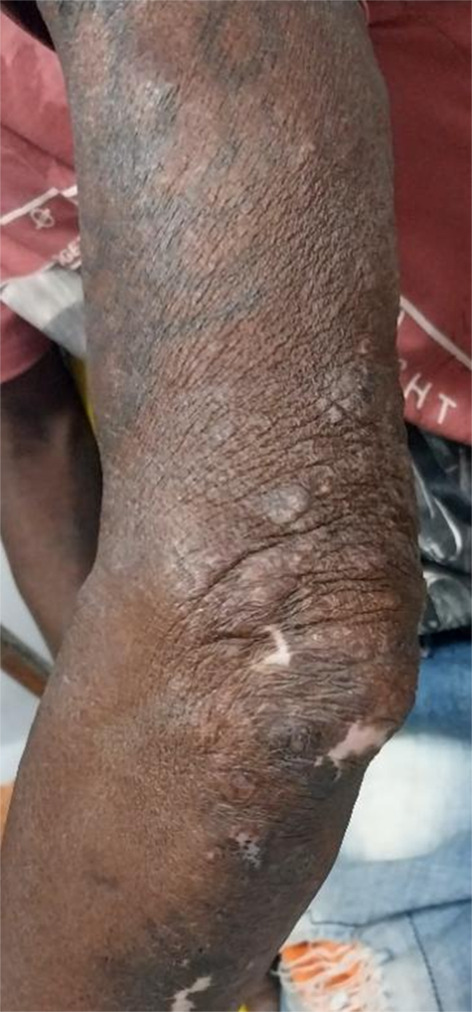
Burns over the elbow

**Fig. 2:**
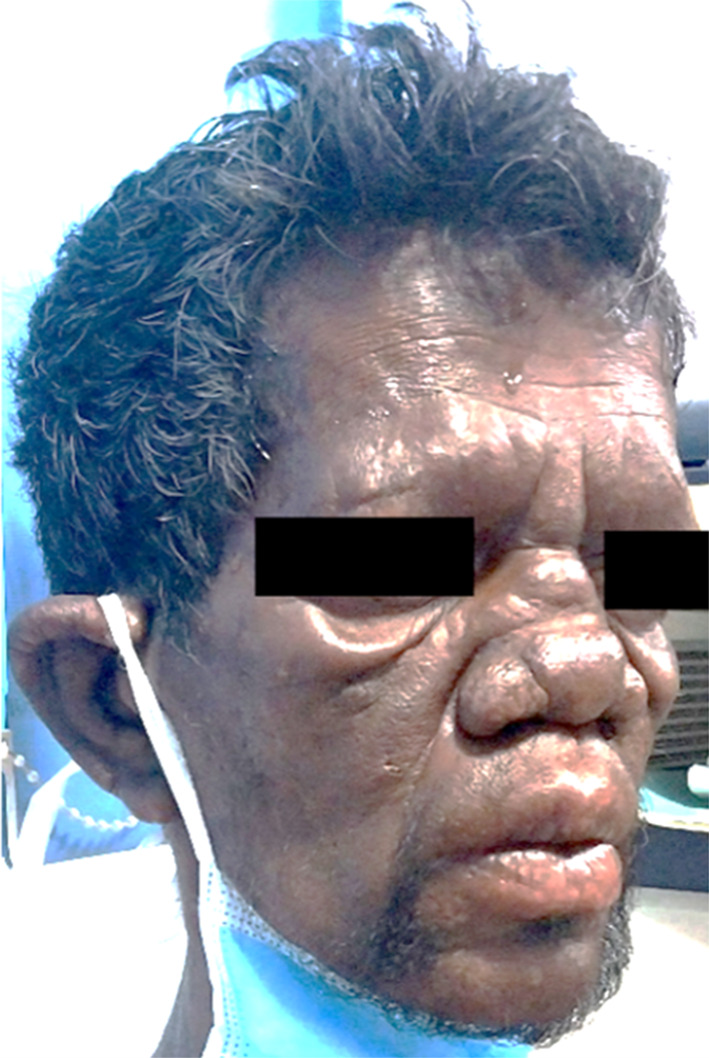
Facial skin and earlobe infiltration with saddle nose deformity and madarosis

On clinical examination, the patient appeared cachexic with bilateral oedema of the hands and feet, pale conjunctiva, and inguinal lymphadenopathy. Wounds were present on both elbows due to occupational silencer burns (Fig. 1). Nodular lesions were noted on the arms, and anaesthetic patches on the inner thighs. Notable signs included facial skin and earlobe infiltration, madarosis, saddle nose deformity (Fig. 2), ichthyosis, and extensive symmetrical skin lesions. Peripheral neuropathy extended to both ankles with numbness in both lower limbs. The patient denied known leprosy contacts and had no fever or joint pain. Past history included high-risk sexual activity, drug use, and a previous stab injury.

Based on clinical features, he was diagnosed with LL. The patient was started on MDT according to the WHO recommended adult MB regimen, and fusidic acid cream was prescribed for wound care while awaiting laboratory results.

### Investigations

Slit skin smears (SSS) microscopy revealed a bacterial index (BI) of + 6 at the ears, eyebrows, and arms and a morphological index (MI) of 15%. Routine blood tests were mostly normal except for low haematocrit level, and the Brewer’s test was negative.

### Follow-up presentation

In June 2025, the patient returned to the Central Leprosy Clinic (CLC), NHSL, reporting worsening symptoms, including erythematous nodules, new macular lesions, persistent lower limb numbness, and difficulty walking. He admitted to discontinuing all medication and defaulting on follow-up visits for more than 18 months. During his initial visit on December 19, 2023, the patient was issued the first monthly blister pack containing the WHO recommended first-line drugs for the adult MB regimen, i.e., rifampicin, clofazimine, and dapsone [16], but he failed to complete the treatment. In this case, the patient’s non-adherence was primarily attributed to limited disease knowledge and poor understanding of the importance of completing MDT.

Examination revealed features consistent with advanced LL, including visible plantar ulcers indicative of Grade 2 disability. On neurological examination, thickening of the left ulnar nerve at the elbow and the left common peroneal nerve at the fibular head was detected on palpation. Sensory examination revealed preserved sensations in both upper and lower limbs. Motor function was intact, with normal eye closure (score 0) and normal motor power in little finger abduction, thumb abduction, wrist extension, and foot dorsiflexion bilaterally. No motor weakness or paralysis was observed. The Eye–Hand–Foot (EHF) score was 0 bilaterally.

Repeat SSS showed a BI of + 6 at the ears and arms and + 5 at the eyebrows, while MI had dropped to 2%. This decline could be due to partial early treatment, although a contributory effect of the host immune response in reducing viable bacilli cannot be excluded. Laboratory investigations revealed anaemia, leucocytosis, and thrombocytosis (Table 1).

**Table 1:** Blood test results at different time points

	Initial visit At diagnosis	Re-visit At re-treatment	Follow up			Units
	15/12/2023	12/06/2025	27/06/2025	18/08/2025	15/09/2025	
SGOT /AST	34	25	24	20	17	(U/l)
SGPT / ALT	13	07	06	09	12	(U/l)
Hb	11.7	10.4	10.5	10.9	11.7	(g/dL)
HCT	35.1	32.2	32.8	34.6	36.3	%
MCV	86.8	78.6	85.0	85.0	91.4	(fL)
G6PD	Negative	Negative	-	-	-	
S. Creatinine	0.66	0.63	-	-	-	(mg/dL)
FBS	-	103.3	-	103.0	-	(mg/dL)
WBC	11.36	14.70	17.03	-	14.51	(10^3^ µL)
Neutrophil count	6.88	7.53	8.47	-	8.15	(10^3^ µL)

### Management

A biopsy was obtained from the nodular lesions at left elbow exclusively for molecular identification of the causative organism using polymerase chain reaction (PCR). Histopathological examination was not pursued, as SSS microscopy had already confirmed the presence of the organism. In routine laboratory practice, histopathological confirmation is not undertaken when SSS findings are diagnostic.

**Fig. 3:**
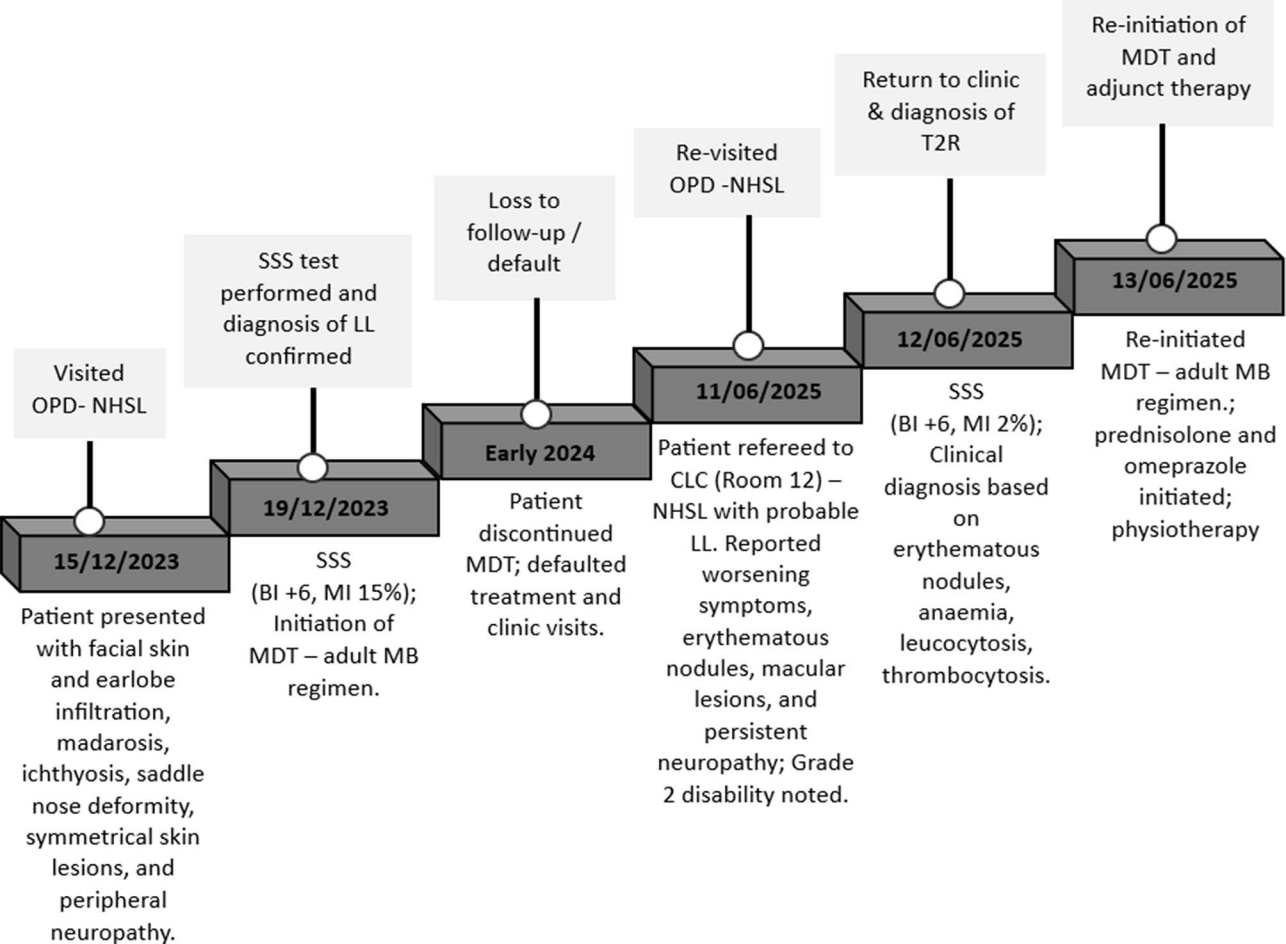
Clinical timeline

MDT was re-initiated on June 13, 2025, using the adult MB regimen (WHO-MDT-MB) recommended by the WHO. On the first day of each 4-week cycle, the patient received a supervised dose of rifampicin 600 mg, clofazimine 300 mg, and dapsone 100 mg. This was followed by daily self-administered doses of clofazimine 50 mg and dapsone 100 mg for the next 28 days [17]. Dapsone may be withheld in patients with moderate to severe anaemia, which is common in chronic leprosy. The treatment is supplied as monthly blister packs, and the patient is expected to complete a total of 12 packs over an 18-month period. The patient was subsequently diagnosed with T2R and was started on oral prednisolone 30 mg daily with omeprazole 20 mg. He also received physiotherapy and foot care under specialist supervision. Monthly clinic follow-up was scheduled, and no adverse drug reactions or complications were reported during subsequent visits (Fig. 3).

Following the diagnosis of T2R, the Public Health Inspector (PHI) for the Mattakkuliya area was notified to initiate contact tracing and community follow-up. The PHI confirmed that the patient was living alone, and no household or close contacts were identified. The patient continues to attend monthly clinic visits for ongoing clinical and treatment monitoring.

## Discussion and conclusion

Leprosy is a chronic spectral disease, and in untreated or inadequately treated cases it can progressively lead to extensive cutaneous, neural, and systemic involvement. The patient in this case presented with advanced LL, characterized by multiple symmetrical skin lesions, thickened peripheral nerves, and Grade 2 disability. This advanced presentation likely reflects delayed diagnosis and interruption of MDT, highlighting the continued challenge of late detection in leprosy control programs.

During the course of the disease, the patient developed T2R, including ENL, an immune complex–mediated complication commonly observed in patients with LL who have high bacillary loads [14]. T2R may occur during MDT, after completion of treatment, or occasionally de novo due to *M. leprae* antigen–antibody immune complex formation [18, 19]. In this case, the patient presented with typical clinical and laboratory features of T2R, including nodular lesions, anaemia, and elevated white blood cell and platelet counts. Additional systemic features such as peripheral oedema, anaemia of chronic disease, and lymphadenopathy further reflected the chronic and inflammatory nature of advanced MB disease and the associated reactional state [20].

The patient’s BI remained high despite partial early treatment, and the MI declined from 15% to 2%, reflecting persistence of degenerated bacilli. Such antigenic remnants can trigger immune complex-mediated inflammation, contributing to T2R even when viable bacilli are reduced [21]. Historically, T2R were more common during the era of dapsone monotherapy, but their prevalence has declined since the introduction of WHO-recommended MDT, partly due to clofazimine’s anti-inflammatory effects [22]. In the present case, treatment default cannot be attributed solely to the occurrence of T2R.

MDT has markedly improved leprosy management by shortening treatment duration and reducing reactions, bringing outcomes close to a cure level. However, WHO programmes and leprologists have long emphasized that early diagnosis is essential to prevent long-term nerve damage, disabilities, and deformities associated with untreated disease. Despite extensive information, education, and communication (IEC) efforts, delayed or missed diagnosis remains a major challenge in leprosy control, as demonstrated in this case where prolonged undiagnosed disease progressed to advanced LL with significant complications.

Structured and targeted history taking at the first contact is essential, as patients with delayed presentation may have received previous treatments without awareness of the diagnosis or medications. Adequate counselling and patient education regarding the importance of completing MDT and potential complications are crucial to improve adherence and reduce disability.

Non-adherence can result from inadequate counseling, limited access to specialized services, drug unavailability, or adverse effects. Addressing these patient and program related barriers is essential to ensure treatment completion, prevent disease progression, and reduce complications.

Barriers to treatment adherence in this patient included social isolation, limited disease knowledge, misconceptions, and lack of family or social support, which led to default after the initial visit. These factors are recognized contributors to non-compliance and delayed treatment, increasing the risk of advanced disease and reactional episodes [23–25]. Additional socioeconomic constraints, such as loss of daily wages and travel difficulties, likely worsened adherence [26]. Family support promotes MDT completion through positive reinforcement [27, 28], and in this case, the patient’s solitary living situation likely compounded the problem.

Management of T2R included corticosteroid therapy with prednisolone [29] to control inflammation and prevent nerve damage [30], combined with regular follow-up to monitor reactions, nerve function, treatment tolerance, and early disability. Close follow-up prevents long term deformities and enables timely interventions, ideally coordinated by field-level PHIs [31]. In this case, the local PHI traced the patient and his contacts, confirming he lived alone with no household or close contacts.

A notable aspect of this case is the prolonged period during which the patient remained undiagnosed, eventually presenting with advanced LL despite improved community awareness, effective MDT, and improved healthcare facilities. Delayed diagnosis in such situations often reflects missed opportunities for early detection at general healthcare facilities, where lack of clinical suspicion remains an important barrier. In addition to diagnostic delay, treatment default may result from multiple patient and program related factors including inadequate counseling at diagnosis, limited patient understanding of the disease, poor access to specialized leprosy services, drug unavailability, adverse drug reactions, and socioeconomic barriers such as travel difficulties or loss of daily wages.

These challenges highlight the need to strengthen general health services through periodic training of healthcare providers, maintain a high index of clinical suspicion, improve referral mechanisms, and enhance coordination between field-level healthcare workers and program managers. Strengthening patient counseling, adherence support, and community-based follow-up through public health programmes is also essential to reduce treatment interruption, ensure early detection of complications, and improve overall leprosy control.

This case illustrates how delayed diagnosis and treatment interruption in LL can lead to severe disease progression and immune-mediated complications such as T2R. It emphasizes that effective leprosy control depends not only on the availability of MDT but also on early case detection, strong clinical suspicion at primary healthcare level, appropriate patient counseling, and sustained follow-up. Strengthening health system capacity, improving referral pathways, and ensuring coordinated field-level surveillance are essential to support treatment adherence and timely management of reactions. The key takeaway from this case is that early recognition of leprosy, uninterrupted MDT, and continuous programmatic support are critical to preventing reactions, reducing disability, and limiting disease transmission.

## Data Availability

Not applicable.

## Abbreviations

MDT: Multidrug therapy
WHO: World Health Organization
AFB: Acid-fast bacilli
PB: Paucibacillary
MB: Multibacillary
TT: Tuberculoid
LL: Lepromatous leprosy
T2R: Type 2 reaction
ENL: Erythema nodosum leprosum
NHSL: National Hospital of Sri Lanka
SSS: Slit skin smears
BI: Bacterial index
MI: Morphological index
CLC: Central Leprosy Clinic
EHF: Eye–Hand–Foot
PCR: Polymerase chain reaction
PHI: Public Health Inspector
IEC: Information, Education, and Communication

## References

[1] Han XY, Sizer KC, Velarde-Félix JS, Frias-Castro LO, Vargas-Ocampo F. The leprosy agents *Mycobacterium lepromatosis* and *Mycobacterium leprae* in Mexico. Int J Dermatol. 2012;51(8):952–9. 10.1111/j.1365-4632.2011.05414.x. 22788812 10.1111/j.1365-4632.2011.05414.x PMC3397401

[2] Polemarches T, Faber WR, Menke H, Rutten V, Pieters T. Reservoirs and transmission routes of leprosy; A systematic review. PLoS Negl Trop Dis. 2020;14(4):e0008276. 10.1371/journal.pntd.0008276. 32339201 10.1371/journal.pntd.0008276 PMC7205316

[3] Mawardi P. Leprosy: the ancient and stubborn disease. Curr Top Trop Emerg Dis Travel Med. IntechOpen; 2018. 10.5772/intechopen.79984.

[4] Alrehaili J, Leprosy, Classification. Clinical Features, Epidemiology, and Host Immunological Responses: Failure of Eradication in 2023. Cureus. 2023;15(9):e44767. 10.7759/cureus.44767. 37809252 10.7759/cureus.44767 PMC10557090

[5] Kar HK, Gupta R. Treatment of leprosy. Clin Dermatol. 2015;33(1):55–65. 10.1016/j.clindermatol.2014.07.007. 25432811 10.1016/j.clindermatol.2014.07.007

[6] Eichelmann K, González González SE, Salas-Alanis JC, Ocampo-Candiani J, Leprosy. An update: definition, pathogenesis, classification, diagnosis, and treatment. Actas Dermosifiliogr. 2013;104(7):554–63. 10.1016/j.adengl.2012.03.028. 23870850 10.1016/j.adengl.2012.03.028

[7] Guidelines for the diagnosis, treatment and prevention of leprosy. New Delhi: World Health Organization, Regional Office for South-East Asia; 2017

[8] Rao PN, Sujai S, Srinivas D, Lakshmi TS. Comparison of two systems of classification of leprosy based on number of skin lesions and number of body areas involved–a clinicopathological concordance study. Indian J Dermatol Venereol Leprol. 2005;71(1):14–9. 10.4103/0378-6323.13779. 16394354 10.4103/0378-6323.13779

[9] Dorilêo GB, Cavalcante LRDS, Lopes JC, Damazo AS. Report of two cases of lepromatous leprosy at an early age. Int J Infect Dis. 2020;101:46–8. 10.1016/j.ijid.2020.09.1448. 32992010 10.1016/j.ijid.2020.09.1448

[10] Chu AC, Greenblatt DT. Dermatologic manifestations of systemic infections. Infect Dis. 2010;140–6. 10.1016/B978-0-323-04579-7.00012-5.

[11] Aluthgamage I, Thilakarathna M, Sathsarani S, Pabodha G, Uthayarajan N, Kottahachchi J, Kahawita I, Deepachandi B. Epidemiological Insights and Current Perspectives of Leprosy in Sri Lanka: A Narrative Review. Bangladesh J Infect Dis. 2025;12(1):159–66.

[12] Vega-Lopez F, Morris-Jones R. Dermatological problems in the tropics. Manson’s Trop Dis. 2024;Fourth Edition:993–1026. 10.1016/B978-0-7020-7959-7.00072-5.

[13] Palaniappan V, Karthikeyan K. Leonine facies and madarosis in lepromatous leprosy. Postgrad Med J. 2022;98(1166):e36. 10.1136/postgradmedj-2021-141061. 34588291 10.1136/postgradmedj-2021-141061

[14] Bhat RM, Vaidya TP. What is New in the Pathogenesis and Management of Erythema Nodosum Leprosum. Indian Dermatol Online J. 2020;11(4):482–92. 10.4103/idoj.IDOJ_561_19. 32832433 10.4103/idoj.IDOJ_561_19 PMC7413435

[15] Perera G, Thilakarathna M, Aluthgamage I, Sathsarani S, Fernando PC, Samaranayake S, Ullah N, Deepachandi B. Unraveling drug resistance in *Mycobacterium leprae*: Exploring genetic mutations to enhance treatment strategies for human leprosy—A narrative review. Int J Microbiol. 2025;2025(1):7204337. 10.1155/ijm/7204337. 41476900 10.1155/ijm/7204337 PMC12752824

[16] Panda PK, Prajapati R, Kumar A, Jana M, Immanuel P, Tanwar P, Wig N. A case of leprosy, erythema nodosum leprosum, and hemophagocytic syndrome: A continuum of manifestations of same agent-host interactions. Intractable Rare Dis Res. 2017;6(3):230–3. 10.5582/irdr.2017.01048. 28944149 10.5582/irdr.2017.01048 PMC5608937

[17] World Health Organization. Guidelines for the diagnosis, treatment and prevention of leprosy. World Health Organization; 2018. https://www.who.int/publications/i/item/9789290226383.

[18] Goulart IMB, Santana MAO, Costa WVTD, Pavelka MM, Dornelas BC. Type 2 leprosy reaction presenting as a monoarthritis post multidrug therapy. IDCases. 2022;27:e01386. 10.1016/j.idcr.2022.e01386. 35036324 10.1016/j.idcr.2022.e01386 PMC8749206

[19] Lubis RD, Yosi A. Overview of leprosy reactions at Universitas Sumatera Utara Medical Faculty Hospitals between 2017 and 2021. Open Access Maced J Med Sci. 2024;12(1):116–21. 10.3889/oamjms.2024.11837.

[20] Dewi DAR, Djatmiko CBP, Rachmawati I, Arkania N, Wiliantari NM, Nadhira F. Immunopathogenesis of Type 1 and Type 2 leprosy reaction: An update review. Cureus. 2023;15(11):e49155. 10.7759/cureus.49155. 38130570 10.7759/cureus.49155 PMC10733783

[21] Tanojo N, Damayanti D, Utomo B, Ervianti E, Murtiastutik D, Cita Rosita Sigit Prakoeswa, Listiawan MY. The demography, clinical characteristics, and white blood analysis of leprosy reactions in multibacillary leprosy: a retrospective study. Berkala Ilmu Kesehatan Kulit Dan Kelamin. 2021;33(3):187–193. 10.20473/bikk.V33.3.2021.187-193.

[22] Celestino IC, Antunes DE, Santos DF, Gimenes VL, de Souza FM, Goulart IMB. Adverse reactions induced by MDT/WHO (Rifampicin+Clofazimine+Dapsone) and ROM (Rifampicin+Ofloxacin+Minocycline) regimens used in the treatment of leprosy: a cohort study in a National Reference Center in Brazil. Front Pharmacol. 2024;15:1346169. 10.3389/fphar.2024.1346169. 38515839 10.3389/fphar.2024.1346169 PMC10955366

[23] Meadows T, Davey G. What factors influence adherence and non-adherence to multi-drug therapy for the treatment of leprosy within the World Health Organisation South East Asia region? A systematic review. Lepr Rev. 2022;93(4):311–31. 10.47276/lr.93.4.311.

[24] Rao PS. A study on non-adherence to MDT among leprosy patients. Indian J Lepr. 2008;80(2):149–54. 19425509

[25] Raju MS, John AS, Kuipers P. What stops people completing multi-drug therapy? Ranked perspectives of people with leprosy, their head of family and neighbours-across four Indian states. Lepr Rev. 2015;86(1):6–20. 26065144

[26] Mushtaq S, Dogra D, Faizi N, Dogra N. Profile of defaulters and pattern of treatment default among leprosy patients at a tertiary care hospital: a 10-year analysis. Indian Dermatol Online J. 2020;11(3):355–60. 10.4103/idoj.IDOJ_393_19. 32695693 10.4103/idoj.IDOJ_393_19 PMC7367569

[27] Susanto T, Dewi EI, Rahmawati I. The experiences of people affected by leprosy who participated in self-care groups in the community: a qualitative study in Indonesia. Lepr Rev. 2017;88(4):543–53.

[28] Hajid SE, Akhbar R, Ansar J, et al. Factors associated with medical treatment compliance among leprosy patients in Gowa district 2015–2016. Indian J Public Health Res Dev. 2019;10(4):899–903. 10.5958/0976-5506.2019.00820.9.

[29] Kahawita IP, Walker SL, Lockwood, Diana NJ. Leprosy type 1 reactions and erythema nodosum leprosum. An Bras Dermatol. 2008;83(1):75–82. 10.1590/s0365-05962008000100010.

[30] Smith WC, Anderson AM, Withington SG, van Brakel WH, Croft RP, Nicholls PG, et al. Steroid prophylaxis for prevention of nerve function impairment in leprosy: Randomized placebo controlled trial (TRIPOD 1). BMJ. 2004;328:1459. 10.1136/bmj.38107.645926.AE. 15159285 10.1136/bmj.38107.645926.AE PMC428511

[31] Ferdinando R, Magodaratna LN, Chandraratne NK, Abeysinghe N, Piyasena D, Wijesinghe MSD. Detailed case analysis of leprosy patients from 2015–2019 in Sri Lanka: Does data quality require improvement? Lepr Rev. 2024;95(2):e2023083. 10.47276/lr.95.2.2023083.

